# A fluorescence-based high-throughput screening method for cytokinin translocation mutants

**DOI:** 10.1186/s13007-020-00676-4

**Published:** 2020-10-07

**Authors:** Mengyuan Zhang, Bingli Ding, Jiangzhe Zhao, Penghong Zhang, Yujia Li, Guodong Yang, Kewei Zhang

**Affiliations:** 1 Institute of Plant Genetics and Developmental Biology, College of Chemistry and Life Sciences, Zhejiang Normal University, Jinhua, 321004 Zhejiang China; 2 Zhejiang Provincial Key Laboratory of Biotechnology on Specialty Economic Plants, Zhejiang Normal University, Jinhua, 321004 Zhejiang China; 3 College of Life Sciences, Shandong Agricultural University, Taian, 271018, Shandong China

**Keywords:** Cytokinin, Translocation, *Arabidopsis* response regulator 5, EMS mutagenesis, Fluorescence reporter

## Abstract

**Background:**

Cytokinins are one kind of phytohormones essential for plant growth, development and stress responses. In the past half century, significant progresses have been made in the studies of cytokinin signal transduction and metobolic pathways, but the mechanism of cytokinin translocation is poorly understood. *Arabidopsis* (*Arabidopsis thaliana*) response regulator 5 (ARR5) is a type-A response factor in cytokinin signaling which is induced by cytokinins and has been used as a reporter gene for the endogenous cytokinins in *Arabidopsis*. Here, we report a fluorescence-based high-throughput method to screen cytokinin translocation mutants using an ethyl methyl sulfone (EMS) mutagenesis library generated with *ARR5*::*eGFP* transgenic plants.

**Results:**

The seedlings with enhanced green fluorescent protein (GFP) signal in roots were screened in a luminescence imaging system (LIS) in large scale to obtain mutants with over-accumulated cytokinins in roots. The selected mutants were confirmed under a fluorescence microscopy and then performed phenotypic analysis. In this way, we obtained twelve mutants with elevated GFP signal in the roots and further found three of them displayed reduced GFP signal in the aerial tissues. Two of the mutants were characterized and proved to be the *atabcg14* allelic mutants which are defective in the long-distance translocation of root-synthesized cytokinins.

**Conclusions:**

We provide a strategy for screening mutants defective in cytokinin translocation, distribution or signaling. The strategy can be adapted to establish a system for screening mutants defective in other hormone transporters or signaling components using a fluorescence reporter.

## Background

Cytokinins are one kind of phytohormones defined by their functions of promoting plant cell division and differentiation [1, 2]. Cytokinins also regulate multiple life processes of plants such as leaf senescence and stress responses [3–5]. They were chemically identified as adenine derivatives and mainly classified as *trans*-zeatin (*t*Z), N^6^-(Δ^2^-isopentenyl) adenine (iP), *cis*-zeatin (*c*Z) and dihydrozeatin (DHZ) types by their multiple side-chains at N^6^ position of the adenine backbone [4].

The biosynthesis of cytokinins in *Arabidopsis* (*Arabidopsis thaliana*) is well understood and the adenosine phosphate-isopentenyltransferases (IPTs) are responsible for the initial step to produce iP nucleotides in the pathway [6, 7]. The *IPT3* expressing in phloem is the major member to produce iP-type cytokinins [8]. The iP-type cytokinins are converted into *t*Z-type cytokinins by two cytochrome P450 monooxygenases CYP735A1 and CYP735A2 which are mainly expressed in the root; the root-synthesized *t*Z needs to be delivered to the shoot for signaling [9]. The biosynthetic sites of cytokinins differing to their action sites suggests that local or long distance transportation processes are required in *Arabidopsis*[4, 10, 11]. The metabolite analysis, transcriptome and grafting experiments showed that the *t*Z and DHZ are mainly produced in the root and moved acropetally while the iP-type cytokinins in the shoot can be moved basipetally or systemically [11–14].

Although the cytokinin translocation pathways have been studied for long time, the molecular mechanisms of cytokinin translocation remain largely unknown. In the early stage, the functional identification of cytokinin transporters was focused on the *Arabidopsis* purine permeases (AtPUPs) and equilibrative nucleoside transporters (ENTs) families, from which several nucleoside transporters were characterized [15, 16]. Biochemical analyses in the heterologous system indicated that some members (AtPUP1, AtPUP2 and OsENT2) of the two families have the capability of transporting cytokinins in yeast [15, 17, 18]. However, the physiological functions of these proteins in plants have not been defined. Physiological experiments showed that SOI33/AtENT8 and its homologous protein AtENT3, as well as rice (*Oryza sativa* L.) PUP4 and PUP7 are involved in the translocation of cytokinins, but the corresponding translocation mechanisms remain to be investigated [19–21].

Breakthroughs have been made in the study of cytokinin translocation mechanisms in recent years, *Arabidopsis* ABCG14 and rice ABCG18 were found as the transporters in the long-distance translocation of cytokinins from root to shoot through the xylem [21–23]. *Arabidopsis* PUP14 is an influx transporter of cytokinins, which lead to a reduction of ligands of cytokinin signal transduction and abnormal embryonic development [24]. However, more transporters involved in cytokinin translocation and the regulators of those transporters remain to be characterized to fully understand the process of cytokinin translocation and signaling.

ARR5 is one of cytokinin response regulators in *Arabidopsis* and its spatiotemporal expression usually reflects the distribution and concentration of cytokinins [25]. Previous reports have shown that AtABCG14 is responsible for the shootward translocation of root-derived cytokinins. *t*Z-type cytokinins are over-accumulated in roots but significantly reduced in shoots of the *atabcg14* mutants, which were visualized by the strength of GUS staining from *GUS* gene expression driven by *ARR5* promoter [22, 23]. Here, we constructed the *ARR5*::*eGFP* vector and used it as a visible reporter to screen mutants defective in cytokinin distribution or signaling in *Arabidopsis*. We performed a mutant screen using a mutagenized *ARR5*::*eGFP* population and gained a batch of mutants with enhanced GFP signal in roots, and finally characterized two *atabcg14* alleles which are defective in cytokinin translocation. The approach to screen cytokinin transporters will greatly contributes to the understanding of the mechanism of cytokinin translocation and signaling.

## Results

### Design of an *ARR5*::*eGFP* reporter-based approach for screening cytokinin translocation mutants

**Fig. 1:**
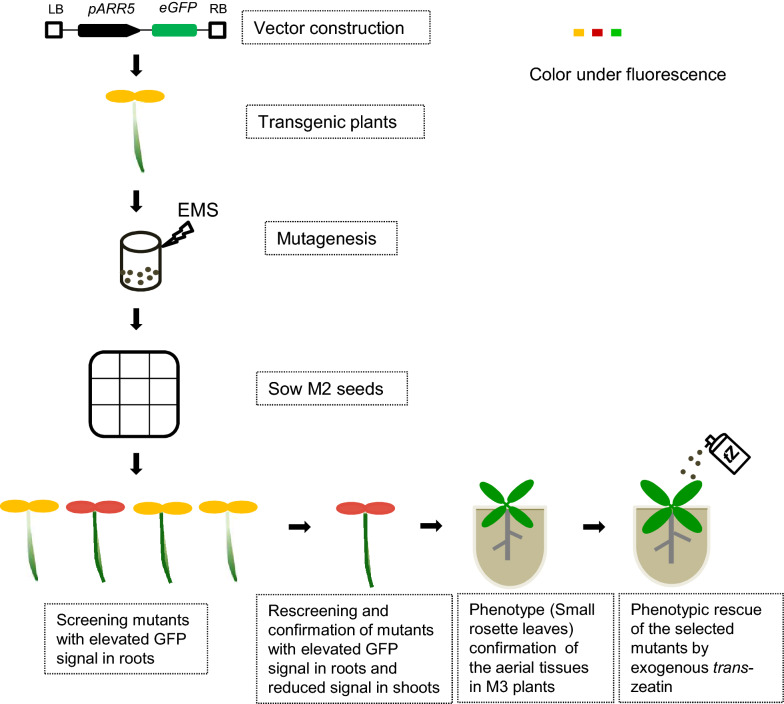
Schematic diagram of the screening system for cytokinin translocation mutants. *ARR5*::*eGFP* (*RCS2*) vector was constructed and *ARR5*::*eGFP* transgenic plants were obtained for mutagenesis by EMS. Mutants were screened by GFP signal observation in whole-plate scale under a luminescence imaging system (LIS) and the phenotypes of the mutants were confirmed individually by fluorescence microscope. The exogenous *t*Z was sprayed into the mutants to further check the mutants defective in shootward long-distance translocation of cytokinins.

To further understand the mechanism of cytokinin shootward translocation, we designed a forward genetic approach for screening cytokinin translocation mutants. A flow chart was presented to screen ethyl methane sulfonate (EMS)-based mutants defective in the shootward translocation of cytokinins (Fig. 1). *t*Z-type cytokinins are synthesized in the root tip of *ARR5*::*eGFP* Col-4 and transported to the shoot through xylem for plant growth and development [11, 26]. Therefore, the root tip of *ARR5*::*eGFP* Col-4 shows a strong GFP signal which was indicated in green color. The overlapping signals of GFP and red autofluorescence of chlorophyll in the leaves were shown in yellow color. The mutants harboring *ARR5*::*eGFP* showed an enhanced GFP signal in roots (shown with green color alongside the root) were screened in a luminescence imaging system (LIS) in large scale. Subsequently, we confirmed the mutants individually under the normal fluorescence microscopy based on the fluorescence signal in roots and shoots. The selected mutants were grown in greenhouse and treated with *t*Z for morphological phenotype analysis to confirm the mutants which are defective in cytokinin long-distance translocation.

### Construction of *ARR5*::*eGFP* reporter vector for screening mutants with over accumulated cytokinins in roots

**Fig. 2:**
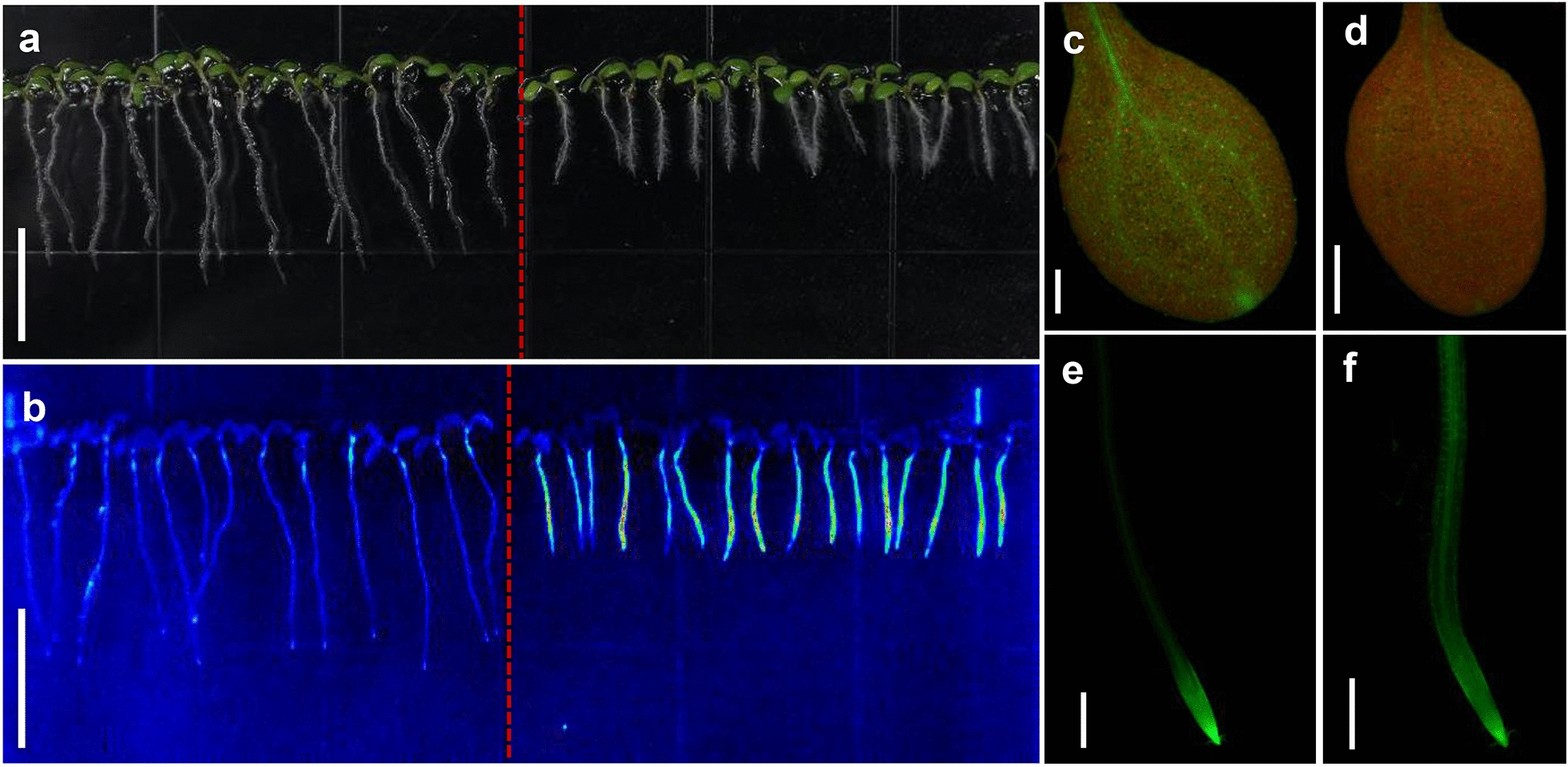
*ARR5*::*eGFP* serves as a cytokinin reporter. **a** Morphological phenotypes of *ARR5*::*eGFP* Col-4 (left) and *ARR5*::*eGFP abcg14* (right). Seeds were sowed on the square plates (10 cm * 10 cm) and grown vertically for 6 days. Scale bar, 1 cm. **b** Fluorescence signal in the roots of transgenic plants of *ARR5*::*eGFP* in Col-4 (left) and *atabcg14* mutants (right) background observed under the LIS. **c**, **d** Fluorescence signal in the true leaves of *ARR5*::*eGFP* Col-4 (left) and *ARR5*::*eGFP abcg14* (right) observed under the fluorescence microscope. Scale bar, 500 μm. **e**, **f** Fluorescence signal in the roots of transgenic plants of *ARR5*::*eGFP* Col-4 (left) and *ARR5*::*eGFP abcg14* (right) observed under the fluorescence microscope. Scale bar, 500 μm

The promoter of *ARR5* was fused to an expression cassette of *eGFP* to be a cytokinin sensitive reporter (Additional file 1: Figure S1). The construct was transferred into Col-4 and *atabcg14*, respectively. The single-copy *ARR5*::*eGFP* transgenic plants in Col-4 and *atabcg14* background were generated. The intensity of GFP signal enables the in situ visualization of cytokinin distribution in transgenic plants (Fig. 2). To screen the mutants in large scale, the GFP signal of *ARR5*::*eGFP* transgenic plants in Col-4 and *atabcg14* background was observed with the LIS. The contrast of the GFP signal intensity in the roots of Col-4 and *atabcg14* suggests that the reporter is suitable for screening the mutants with altered cytokinin distribution and expression in large scale (Fig. 2a, b). Under fluorescence microscopy, the green fluorescence intensity was significantly reduced in the shoot of *atabcg14* compared to the Col-4 (Fig. 2c, d). Strong fluorescence signal was spread in the whole root of *atabcg14* but only in the root tip of Col-4 (Fig. 2e, f), suggesting the results from the LIS are consistent to that from fluorescence microscopy.

### Mutant screening with *ARR5*::*eGFP* reporter

**Fig. 3:**
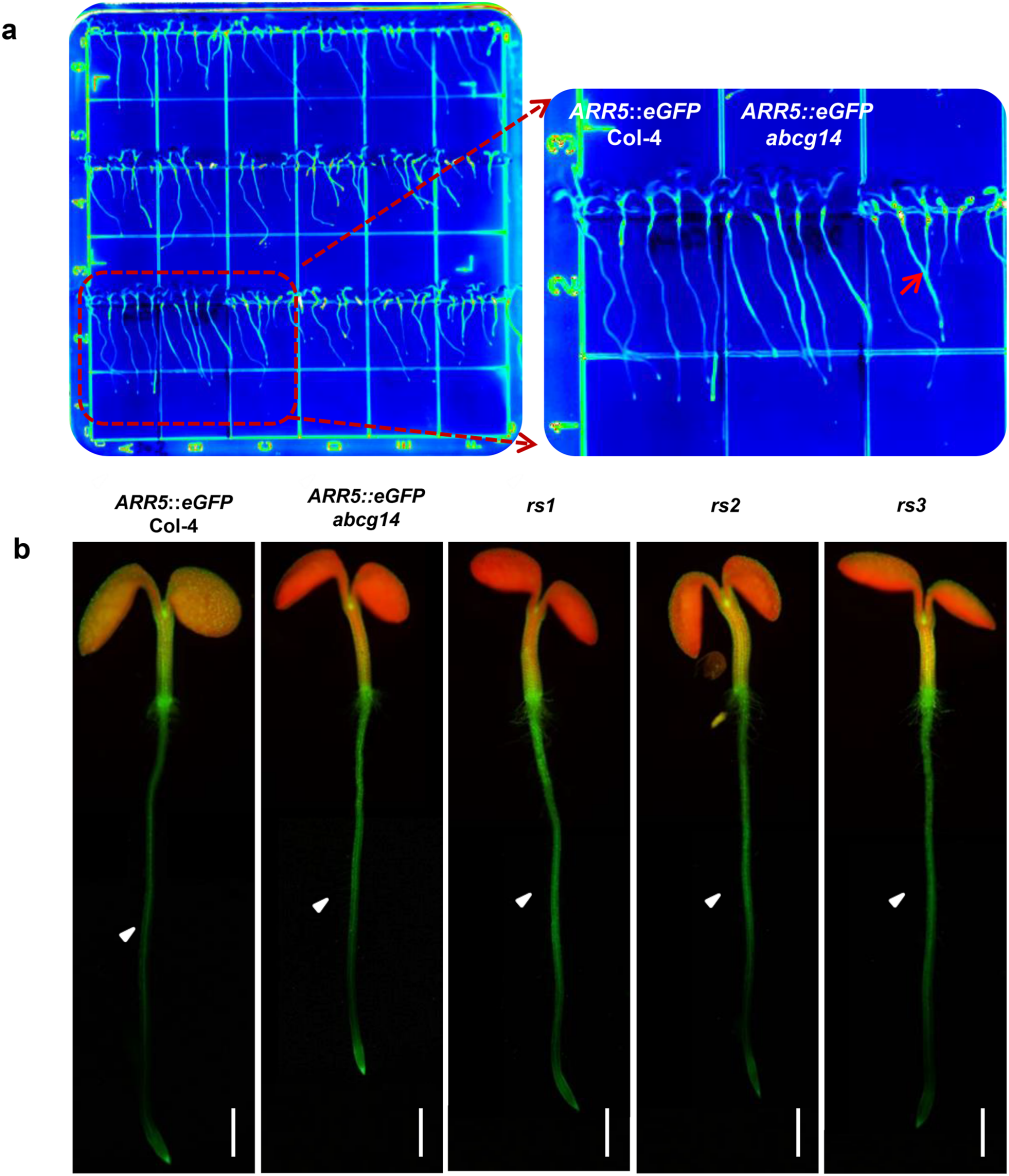
Screening and confirmation of mutants defective in cytokinin translocation. **a** Mutant screening with the elevated GFP signal in the roots. M2 seeds were sowed on the square plates (10 cm * 10 cm) and cultured vertically. 6-DAG mutants were observed and screened with the LIS (left). Red arrow indicates the target mutant (right, ×2 magnification). *ARR5*::*eGFP* Col-4 and *ARR5*::*eGFP abcg14* were used as the controls. **b** GFP signal of *rs1* to *3* at 5-DAG. White triangles pointing to the tissues with changed GFP signal in roots. DAG, days after germination. Scale bar, 1 mm

The seed stock of *ARR5*::*eGFP* transgenic plants in Col-4 background was used for EMS mutagenesis. The M1 seeds in agar liquid were sown in soil by the pipette with tips. The seeds of 50 M1 lines were set as a pool and 300 pools were harvested. About 36,000 to 48,000 M2 seeds from 120 pools were used for mutant screening. We optimized the growth conditions for mutant screening and found that the 1% sucrose medium and 24-h light are the optimal conditions to observe root phenotypes (Additional file 2: Figure S2). Seeds were sowed on the square plates (10 cm * 10 cm) containing 1/2 MS media and cultured vertically for 6–8 days. The GFP signal of M2 seedlings in roots was observed by the LIS for the large-scale screening (Fig. 3a). Mutants with elevated GFP signal in roots were selected, numbered and transplanted into the soil. After the large-scale screening, we obtained 56 preliminary mutants with elevated GFP signal in roots.

### Confirmation of the selected mutants by phenotypic observation

To further confirm the mutants selected in the large-scale screening, we checked the GFP expression in each mutant at M3 generation with fluorescence microscopy. After detailed observation with fluorescence microscopy, we obtained 20 mutants from the 56 preliminary mutants which spread GFP signal in the whole root tissues (except one of them showed an interesting eclipsed GFP signal in the elongation zone of root), suggesting the cytokinin accumulation might occur in the roots of those *atabcg14*-like mutants. We confirmed the phenotypic stability of the mutants at M3 generation and made backcross to *ARR5*::*eGFP* Col-4 to check the separation rate of the mutant phenotype. Finally, we got 12 mutants with stable phenotype and separation rate following Mendel’s law and designed them as *root shining* (*rs*) mutants based on their enhanced GFP signal in roots (Additional file 3: Figure S3).

Among the 12 mutants with elevated GFP signal in roots, three lines of the *rs* mutants exhibited brilliant GFP expression in roots but reduced signal in shoots (Fig. 3b). The *rs1* to *3* were performed detailed phenotypic analysis using *ARR5*::*eGFP* Col-4 and *ARR5*::*eGFP abcg14* as controls. For *ARR5*::*eGFP* Col-4, GFP was expressed mainly in the root pericycle, while it was expressed strongly in both pericycle and epidermis of root in *ARR5*::*eGFP abcg14* (Fig. 2e, f), reflecting cytokinins were stacked in the pericycle and diffused into the epidermis of *atabcg14* roots. Under fluorescence microscopy, the distribution of GFP signal in *rs1* to *3* displayed a similar pattern with *ARR5*::*eGFP abcg14* (Fig. 3b), indicating that *rs1* to *3* may be mutants defective in long-distance translocation of root-synthesized cytokinins.

### Phenotypic analysis of the roots of the selected mutants

**Fig. 4:**
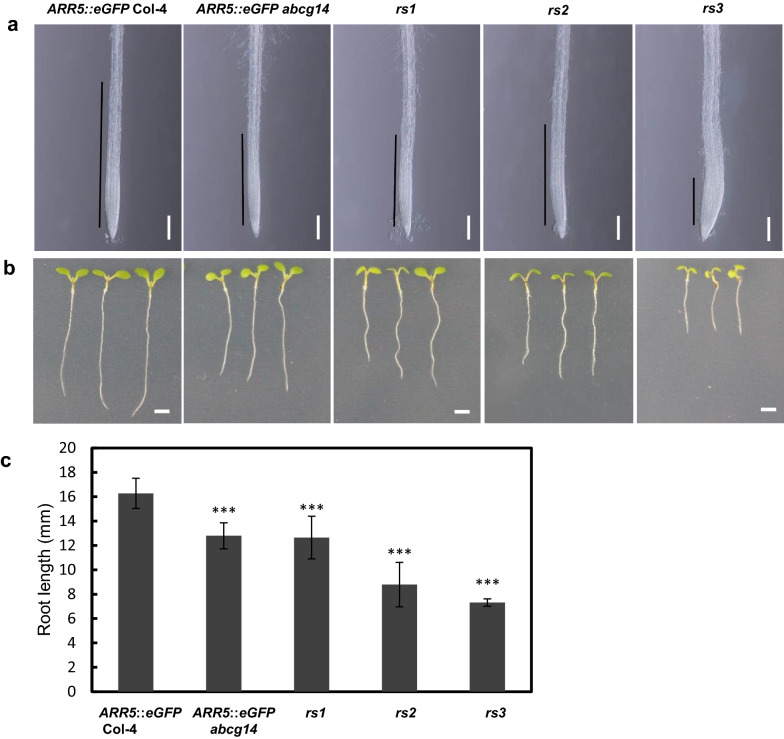
The morphological phenotypes of the roots of *rs* mutants. **a** Phenotypic observation on root meristems of 6-DAG *rs* mutants. Black lines represent the lengths of the meristem and elongation zone. Scale bar, 200 µm; **b** Root phenotypes of 6-DAG *rs* mutants. Scale bar, 2 mm; **c** Quantification of the root lengths of 6-DAG *rs* mutants. Data are mean ± SD (n = 6, biological replicates). Asterisks indicate significant differences compared to *ARR5*::*eGFP* Col-4 (***P < 0.001, Student’s *t*-test)

The changes of cytokinin distribution in the roots and shoots affect the cytokinin signaling which determines multiple development phenotypes, such as rosette leaf size, developmental phase, stem diameter and floral primordia [12, 27, 28]. To verify whether the enhanced GFP signal of *rs* mutants in roots has the genetic effects as cytokinins, we conducted genetic phenotypic observations and analyses of *rs* mutants. Firstly, in order to understand the effect of enhanced GFP signal on root development, we observed the meristem structures of roots in *rs* mutants. As previous reports, over-accumulated cytokinins reduced the activity of root apical meristems (RAMs) and inhibited root elongation [29–31]. Similar to *ARR5*::*eGFP abcg14*, *rs1* to *3* which have enhanced GFP signal in roots showed significant reduction in size of the root meristem and elongation zone (Fig. 4a). In consistent with the effects of cytokinin accumulation on root growth, *rs1* to *3* have shorter roots compared to Col-4 (Fig. 4b, c). These results showed that the enhanced GFP signal of roots in *rs1* to *3* was able to represent the elevated levels of cytokinins which inhibited root elongation by affecting the meristem activity.

### Phenotypic analysis of the shoots of the selected mutants

**Fig. 5:**
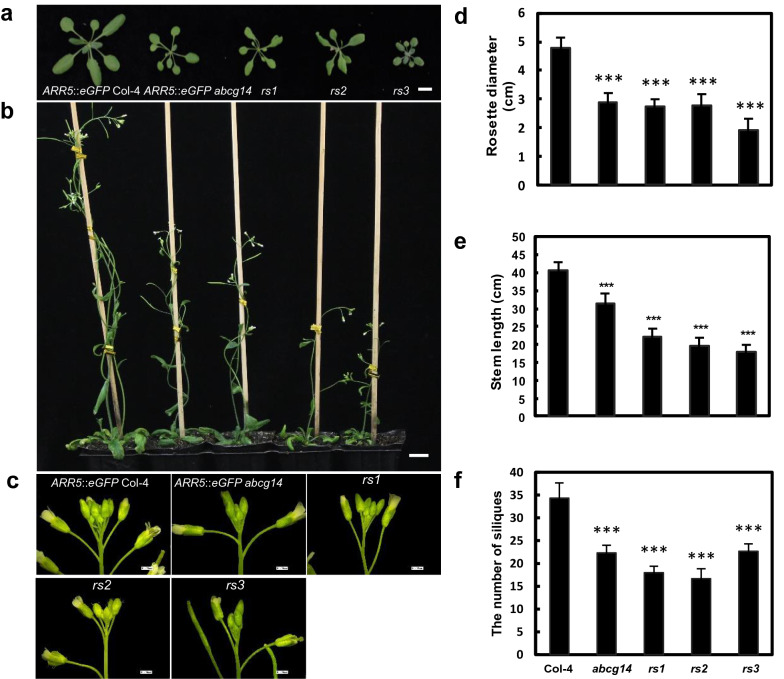
Morphological phenotypes in the aerial tissues of *rs* mutants. **a**, **d** Morphological phenotype and statistical diameters of rosette leaves of 21-DAG *rs* mutants. Scale bar, 1 cm; **b**, **e** Morphological phenotype and plant heights of *rs* mutants at 35-DAG and 50-DAG, respectively. Scale bar, 2 cm; **c**, **f** Inflorescence and silique numbers of *rs* mutants at 35-DAG and 50-DAG, respectively. Scale bar, 100 µm; Data are mean ± SD (n = 6, biological replicates); ***P < 0.001 (Student’s *t*-test); DAG, days after germination

While the GFP signal was increased in the roots, it was reduced in the shoots of *rs1* to *3* (Fig. 3b). Cytokinins have been previously reported to inhibit the activity of RAMs, but promote the activity of shoot apical meristems (SAMs) [27]. *t*Z-type cytokinins are known to be synthesized in root tip and transported to the shoot to regulate the rosette area in *Arabidopsis* [32]. Consistent with the reduced GFP signal in the shoots of *rs1* to *3*, cytokinin-deficient phenotypes were exhibited in the aerial tissues of *rs1* to *3*, such as dwarf and reduction of floral primordium (Fig. 5a–c). In addition, the rosette leaf size and plant height of *rs1* to *3* were measured. As expected, the rosette leaf diameters, stem lengths and the number of siliques of *rs* mutants were decreased significantly (Fig. 5d, e, f). Therefore, the intensity and distribution of GFP fluorescence signal are consistent with the plant morphological phenotype.

### Exogenous *trans*-zeatin treatment rescued some of the selected mutants to wild type

**Fig. 6:**
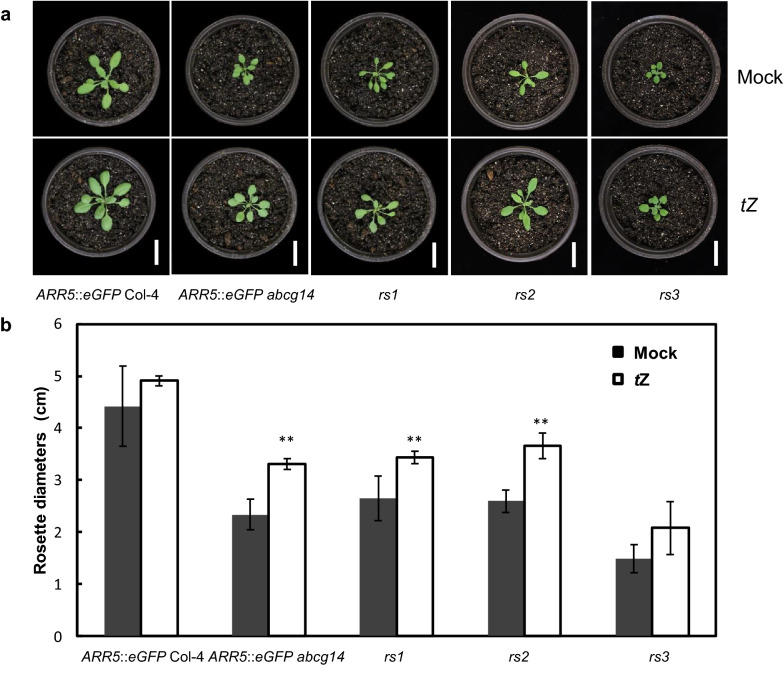
Exogenous application of *t*Z rescued the phenotypic defects of *rs* mutants. **a** Morphological phenotypes of *rs* mutants and a positive control (*ARR5*::*eGFP abcg14*) after 10-day treatment with 0.5% methanol (Mock) or 5 μM *t*Z. Scale bar, 3 cm. **b** Rosette leaf diameters of *rs* mutants and controls at 20-DAG. DAG, days after germination. Data are mean ± SD (n = 6, biological replicates); Asterisks indicate significant differences compared to Mock (**P < 0.01, Student’s *t*-test)

*rs1* to *3* showed a genetic phenotype similar to *atabcg14* mutants harboring *ARR5*::*eGFP*, so we hypothesized that *rs1* to *3* are mutants defective in shootward translocation of cyotokinins. Exogenous *t*Z treatment can induce cytokinin signaling in the shoots of cytokinin translocation mutants and rescue the growth retardation phenotype to wild-type [32]. Therefore, we performed the exogenous *t*Z treatment experiment to check whether *rs1* to *3* are mutants defective in cytokinin translocation or signaling. After *t*Z treatment for 10 days, the deficiency of the rosette leaf expansion of *rs1* to *3* was obviously rescued by *t*Z respectively (Fig. 6a, b). It proved that the development defects of *rs1* to *3* in shoots were caused by cytokinin-deficiency, verifying they are cytokinin translocation mutants.

### Map-based cloning of *rs* mutants

**Fig. 7:**
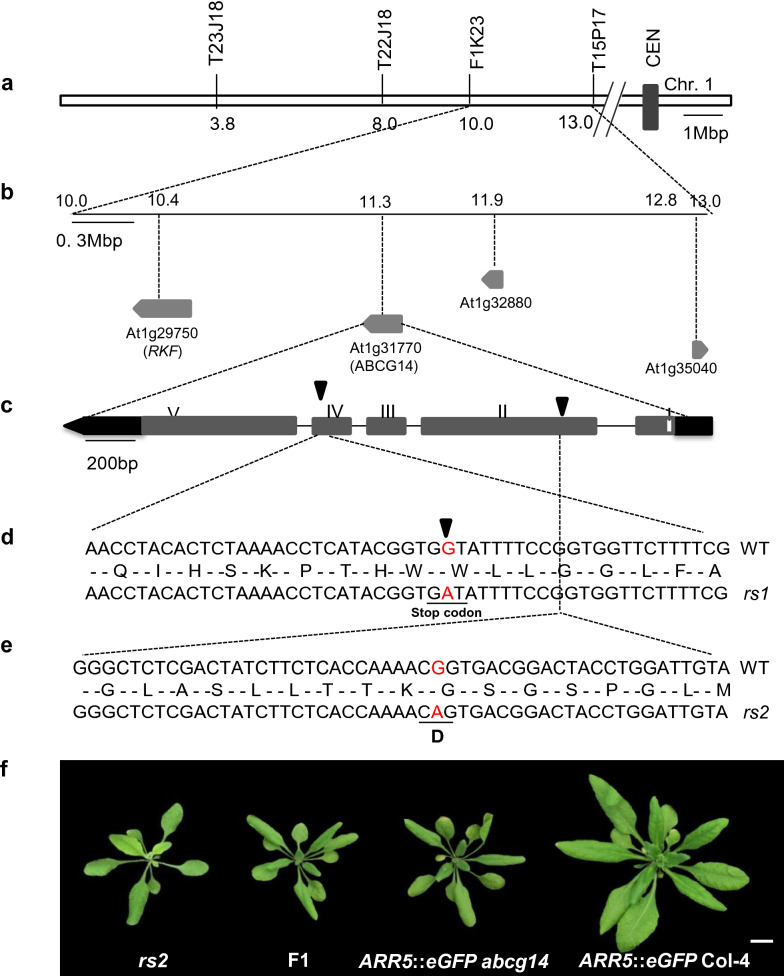
Gene cloning of *rs1* and *rs2*. **a** The genetic map position of the *rs1* and *rs2*. Molecular markers (top) and their physical positions (bottom) were indicated. CEN represent the centromere. **b** Four candidate genes of *rs1* were presented. Numbers indicate the physical position in M bp of candidate genes. Grey arrows indicate genes direction and accession numbers (below). **c** Schematic of *ABCG14* genetic structure. The genomic DNA of *ABCG14* contains 5 exons. Black arrows indicate the position of mutation in the exons. **d** A point mutation (G → A) occurred to *rs1* which generated a stop codon in protein-coding sequence is underlined. **e** A point mutation (G → A) occurred to *rs2* and caused a mutation from Glycine to Aspartic acid in amino acid sequence. **f** Conformation of *rs2* as an *atabcg14* allele by crossing *rs2* with *ARR5*::*eGFP abcg14*. Scale bar, 1 cm

Map-based cloning showed that the mutations of *rs1* and *rs2* are both on the chromosome 1 where *AtABCG14* is located (Fig. 7a, b). Since the phenotypic similarities of *rs1*, *rs2* and *atabcg14*, the genomic DNA of *AtABCG14* gene was sequenced in *rs1* and *rs2*. The results showed that a point mutation occurred to *AtABCG14* in *rs1* which converted the 539th amino-acid from Tryptophan to a stop codon (Fig. 7d); meanwhile, the *AtABCG14* of *rs2* carrying a novel mutation allele, by which the 162th amino-acid Glycine mutated into an Aspartic acid in the nucleotide binding domain (Fig. 7e). The genetic hybridization result confirmed *rs2* is a novel *atabcg14* allele (Fig. 7f). The successful characterization of the two novel *atabcg14* alleles suggested that we have developed a powerful tool for screening the cytokinin translocation mutants.

## Discussion

Cytokinins play essential roles in plant growth, development and stress responses and have promising applications in molecular breeding [33–35]. The sites of cytokinin biosynthesis and perception are generally multiple, so the short-range and long-distance translocation are required in *Arabidopsis*. Compared to the well-known transporters of other hormones such as auxin and abscisic acid (ABA), the knowledge of cytokinin transporters remains poor. The yeast-based biochemical approach suggested that AtPUP1, AtPUP2 and OsENT2 are cytokinin transporters [15, 17, 18], but the physiological functions of these proteins in plants have not been clarified. On the other side, the genetics-based approach revealed the ENT8/SOI33, ABCG14 and PUP14 are committed to cytokinin translocation in *Arabidopsis* [19, 22–24], suggesting that genetic approach is powerful for characterization of cytokinin transporters. As is well known, the forward genetics provides an approach for screening mutants with specific phenotype in genome-wide. The mutant screening approach we presented here is low-cost and convenient for characterizing the novel cytokinin transporters or the novel regulators of some well-known cytokinin transporters.

ARR5 is one of the cytokinin response regulators whose promoter was fused to *GUS* gene to be used as a cytokinin reporter widely [25]. We fused the promoter of *ARR5* to *eGFP* gene to form a reporter *ARR5*::*eGFP* to check the concentration and distribution of cytokinins in living plants of *Arabidopsis*. The distribution of GFP signal in the transgenic plants of *ARR5*::*eGFP* can be checked in large scale by the LIS for non-damage screening (Fig. 2b). By this way, a mutant library were constructed and screened for cytokinin translocation mutants in large-scale. We obtained a batch of mutants with enhanced GFP signal in roots by the novel screening method. *rs1* to *3* presented a genetic phenotype similar to *ARR5*::*eGFP abcg14* and we speculated that they are mutants with over-accumulated cytokinins in the roots (Figs. 4, 5). Phenotypic recovery of *rs1* to *3* by exogenous *t*Z treatment proved that *rs1* to *3* are mutants defective in cytokinin shootward translocation (Fig. 6). The characterization of *rs1* and *rs2* as novel alleles of *atabcg14* suggested that the screening system is powerful for screening the mutants of cytokinin shootward translocation (Fig. 7). *TCSn::eGFP* is a robust synthetic sensor to monitor the transcriptional output of the cytokinin signaling network *in planta*, which is more sensitive than the *ARR5::eGFP* [36]. The establishment of a similar screening procedure using *TCSn::eGFP* reporter would be more effective for mutant screening. Regarding this point, this procedure can be adapted for screening the mutants defective in cytokinin short-range translocation or signaling. In addition, the strategy can be used for a reference to establish a system for screening mutants defective in other hormone transporters or signaling components using a fluorescence reporter.

## Conclusions

An *ARR5::eGFP* reporter based forward genetic approach was set up to screen mutants defective in cytokinin shootward translocation. The successful screening of two *atabcg14* mutant alleles confirmed the efficiency and reliability of the high-throughput screening strategy. The approach may be adapted for screening mutants defective in rootward or short-range cytokinin transportation, or in cytokinin signaling.

## Methods

### Plant materials and growth conditions

*Arabidopsis thaliana* ecotypes *Columbia*-*4* (Col-4) and *Landsberg* (L*er*) were respectively used as the wild-type lines in the experiments. The *atabcg14* mutant was described previously [22]. Mutagenic seeds were sown on the square plates (10 * 10 cm) containing half-strength Murashige and Skoog (1/2 MS, Phytotech) media with 0.25% (w/v) phytogel (Sigma) or 0.5% (w/v) Gellan Gus Powder G434 (Phytotech) and 1% (w/v) sucrose (Sigma) and incubated at 4 °C in dark for 3 days. Seedlings were cultured vertically at 22 °C in an illumination incubator (Percival) under a 24-h light growth regime with approximately 60% humidity and 110 µmol m^−1^ s^−1^ light intensity. Then, seedlings were transplanted into soil and grown in a growth chamber at 22 °C and a long-day (16-h light/8-h dark) growth regime with approximately 60% humidity and 120 µmol m^−1^ s^−1^ light intensity.

### Vector construction and transformation

To generate the report vector, the promoter of *ARR5* was cloned by gene-specific primers *ARR5*-P1 and P2 (Additional file 4: Table S1). A 1606-bp product was constructed into *pSAT6*-*35S*::*eGFP* with Age I and Nco I (TaKaRa). Then, the vector was digested with PI-PspI (NEB) and the released fragment was constructed into *pPZP*-*RCS2* to generate the construct *ARR5*::*eGFP* [37]. The binary vector was transferred into GV3101 (*A.tumefaciens*) for genetic transformation by floral dipping method [38].

### The screening of single-copy transgenic plants

*ARR5*::*eGFP* transgenic plants were obtained by screening the seedlings of T1 generation with 1/2 MS media containing 15 µg/mL Hygromycin (Hyg) and 50 µg/mL Timentin (Tim). The single-copy insertion lines were identified by the segregation ratio with 3/4 Hyg resistance of T2 seedlings. The homozygous single-copy lines of *ARR5*::*eGFP* transgenic plants were used for the study.

### EMS mutagenesis and mutant screening

EMS mutagenesis was performed using a homozygous single-copy population of *ARR5*::*eGFP* Col-4 as the stock. The population was treated with 0.2% EMS solution for 15 h and washed by tap water and sowed in soil following a protocol previously described [39]. The seeds from 50 M1 plants were harvested and pooled in one tube. 300–400 seeds of each tube (pool) were used for the screening. The M2 population was sowed on the square plates containing 1/2 MS media and cultured vertically for 6–8 days, with a few seeds of transgenic plants of *ARR5*::*eGFP abcg14* as the positive control and *ARR5*::*eGFP* Col-4 as the negative control. The LIS (Tanon 6600) in fluorescent observation condition was used to observe the GFP signal in M2 seedlings and performed the initial screening. The mutants with enhanced GFP signal in roots compared to *ARR5*::*eGFP* Col-4 were chosen and transplanted into the soil. According to the genetic phenotypes of *atabcg14*, phenotypic observation and analysis of the aerial tissues of the mutants were performed. The mutants identified from the screening were named as *root shining* (*rs*) mutant. Twelve lines selected in this approach were designed as *rs1* to *12*.

### Fluorescence signal observation

Mutants were screened by checking the intensity of fluorescence signal in roots. The initial fluorescence observation of mutants was performed in whole-plate scale by the LIS containing a fluorescent module. The fluorescence signal of mutants was confirmed by using the microscope (Olympus BX53) with fluorescence unit (Olympus U-RFL-T).

### Morphology phenotypic analysis of roots and aerial tissues

The roots of 6-DAG (days after germination) mutant seedlings were photographed (Canon EOS 60D) and measured (Image J). The root meristems of 6-DAG mutant seedlings were observed and photographed by the stereomicroscope (Zeiss SteREO Discovery.V12). The rosette leaves of 21-DAG mutants were photographed (Canon EOS 60D) and performed diameter measurement (Image J). The morphological phenotypes of 35-DAG mutants were observed (Canon EOS 60D) and plant heights at 50-DAG were measured (Image J). The inflorescences of 35-DAG mutants were observed (KEYENCE VHX-5000 3D ultra-deep field microscope) and the number of siliques was counted at 50-DAG.

### Exogenous *t*Z treatment

For *t*Z complementary experiment, 10-DAG seedlings of the selected mutants and *atabcg14* mutant (a positive control) were daily sprayed with 5 μM *t*Z (Sangon Biotech, Shanghai, China) dissolved in 0.5% methanol at 10 AM. The same solution without *t*Z was used as the mock treatment. After 10 days of treatment, rosette leaf diameters of the mutants and the controls were measured. The statistical analysis of the differences between samples was calculated using the Student’s *t* test.

### Map-based cloning and DNA sequencing

For map-based gene cloning of *rs* mutants, we employed the 25 pairs of markers from http://amp.genomics.org.cn/ [40]. The *rs* mutants were crossed with *ARR5*::*eGFP* L*er* and the F1 plants were self-fertilized to yield a homologous recombined population. Mutants isolated from the F2 plants were analyzed for map-based cloning to identify the mutation sites. The *AtABCG14* DNA fragments were sequenced using primers *AtABCG14*-P1 and P2 in Additional file 4: Table S1.

## Supplementary information

**Additional file 1: Figure S1.** Construction of *ARR5*::*eGFP* reporter vector. The promoter of *Arabidopsis RR5* was cloned and constructed into the plant expression vector (*RCS2*) to drive free *GFP* expressing. The map was prepared by SnapGene

**Additional file 2: Figure S2.** The growth conditions were optimized for mutant screening. **a** Seedlings of *ARR5*::*eGFP* Col-4 and *ARR5*::*eGFP abcg14* showed discriminating phenotypes in culture sucrose at 1%, 2% and 3%. The effects of long day (LD, 16-h light/8-h dark) and all light (AL, 24-h light) growth regimes on root growth of *ARR5*::*eGFP abcg14* were demonstrated. Scale bar, 3 mm. **b** Root lengths of *ARR5*::*eGFP* Col-4 and *ARR5*::*eGFP abcg14* at 6-DAG. Data are mean ± SD (n = 6, biological replicates); Asterisks indicate significant differences compared to *ARR5*::*eGFP* Col-4 (**P < 0.05, **P < 0.01, ***P < 0.001, Student’s *t* test)

**Additional file 3: Figure S3.** Morphological phenotypes of *rs1* to *rs12*. **a** The rosette leaves of *rs* mutants at 25-DAG. Scale bar,1 cm; **b** GFP signal in the roots of *rs* mutants at 6-DAG. White triangles pointing to the elevated GFP signal in mutants. DAG, days after germination

**Additional file 4: Table S1.** Primers used in this study

## Data Availability

The author responsible for distribution of materials integral to the method is: Kewei Zhang, Email: kwzhang@zjnu.edu.cn.
