# Supplementary material for: A fluorescence-based high-throughput screening method for cytokinin translocation mutants
Source: Plant Methods. 2020 Oct 7;16:134. doi: 10.1186/s13007-020-00676-4 (PMC7539434; doi:10.1186/s13007-020-00676-4)
Supplement: Supplementary file 1 — Additional file 1: Figure S1. Construction of ARR5::eGFP reporter vector. The promoter of Arabidopsis RR5 was cloned and constructed into the plant expression vector (RCS2) to drive free GFP expressing. The map was prepared by SnapGene [file 13007_2020_676_MOESM1_ESM.pdf]

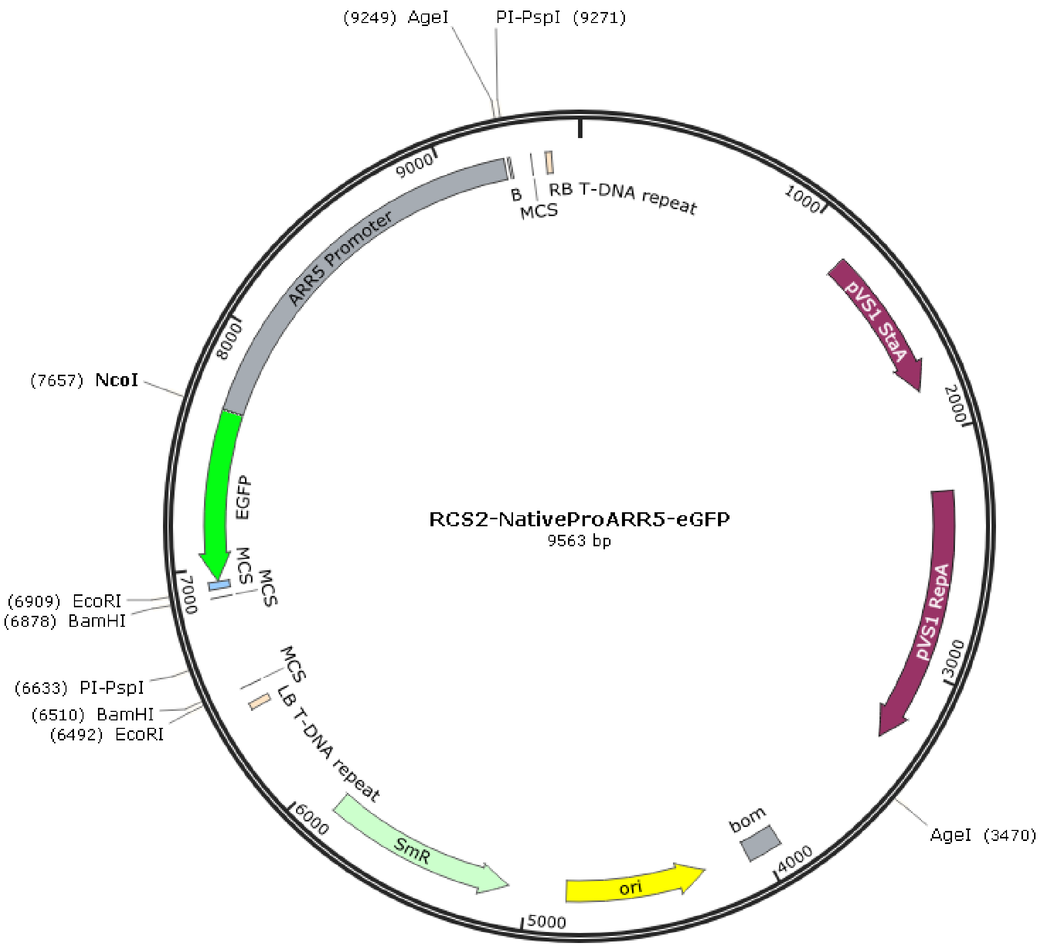

Figure S1 Construction of *ARR5*::*eGFP* reporter vector. The promoter of *Arabidopsis RR5* was cloned and constructed into the plant expression vector (*RCS2*) to drive free *GFP* expressing. The map was prepared by SnapGene.
