# Supplementary material for: A fluorescence-based high-throughput screening method for cytokinin translocation mutants
Source: Plant Methods. 2020 Oct 7;16:134. doi: 10.1186/s13007-020-00676-4 (PMC7539434; doi:10.1186/s13007-020-00676-4)
Supplement: Supplementary file 2 — Additional file 2: Figure S2. The growth conditions were optimized for mutant screening. a Seedlings of ARR5::eGFP Col-4 and ARR5::eGFP abcg14 showed discriminating phenotypes in culture sucrose at 1%, 2% and 3%. The effects of long day (LD, 16-h light/8-h dark) and all light (AL, 24-h light) growth regimes on root growth of ARR5::eGFP abcg14 were demonstrated. Scale bar, 3 mm. b Root lengths of ARR5::eGFP Col-4 and ARR5::eGFP abcg14 at 6-DAG. Data are mean ± SD (n = 6, biological replicates); Asterisks indicate significant differences compared to ARR5::eGFP Col-4 (**P < 0.05, **P < 0.01, ***P < 0.001, Student’s t test) [file 13007_2020_676_MOESM2_ESM.pdf]

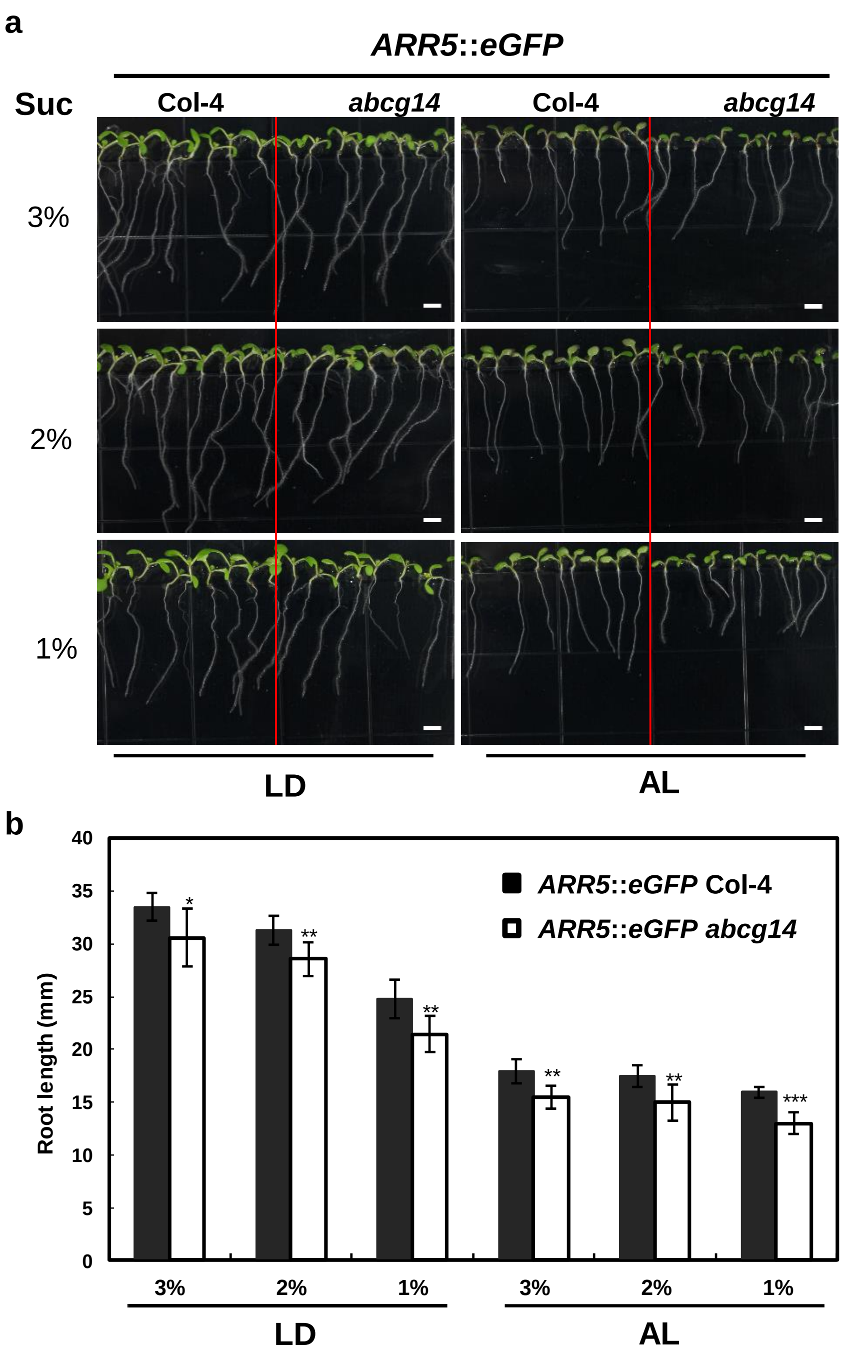

Figure S2 The growth conditions were optimized for screening. **a** Seedlings of *ARR5*::*eGFP* Col-4 and *ARR5*::*eGFP abcg14* showed discriminating phenotypes in culture sucrose at 1%, 2% and 3%. The effects of long day (LD, 16-h light/8-h dark) and all light (AL, 24-h light) growth regimes on root growth of *ARR5*::*eGFP abcg14* were demonstrated. Scale bar, 3 mm. **b** Quantification of root lengths of *ARR5*::*eGFP* Col-4 and *ARR5*::*eGFP abcg14* at 6-DAG. Data are means±SD (n=6, biological replicates); Asterisks indicate significant differences compared to *ARR5*::*eGFP* Col-4 (*P < 0.05, **P < 0.01, ***P < 0.001, Student's *t*-test).
