# Supplementary material for: A fluorescence-based high-throughput screening method for cytokinin translocation mutants
Source: Plant Methods. 2020 Oct 7;16:134. doi: 10.1186/s13007-020-00676-4 (PMC7539434; doi:10.1186/s13007-020-00676-4)
Supplement: Supplementary file 3 — Additional file 3: Figure S3. Morphological phenotypes of rs1 to rs12. a The rosette leaves of rs mutants at 25-DAG. Scale bar,1 cm; b GFP signal in the roots of rs mutants at 6-DAG. White triangles pointing to the elevated GFP signal in mutants. DAG, days after germination [file 13007_2020_676_MOESM3_ESM.pdf]

a

b

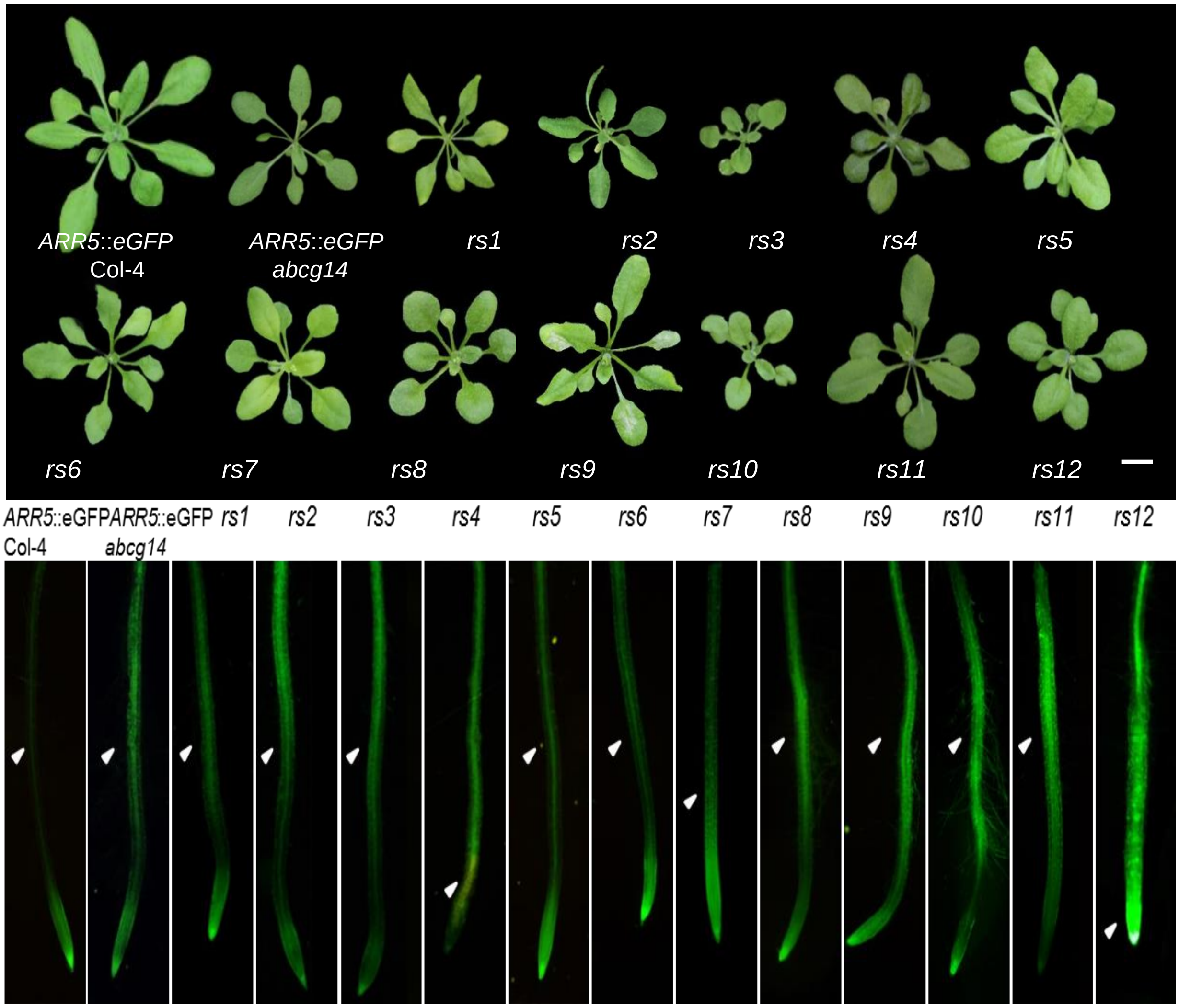

Figure S3 Morphological phenotypes of *rs1* to *rs12*. **a** The rosette leaves of *rs* mutants at 25 DAG. Scale bar,1 cm; **b** GFP signal in the roots of *rs* mutant at 6 DAG. White triangles pointing to the elevated GFP signals in mutants.
