# Supplementary material for: A fluorescence-based high-throughput screening method for cytokinin translocation mutants
Source: Plant Methods. 2020 Oct 7;16:134. doi: 10.1186/s13007-020-00676-4 (PMC7539434; doi:10.1186/s13007-020-00676-4)
Supplement: Supplementary file 4 — Additional file 4: Table S1. Primers used in this study [file 13007_2020_676_MOESM4_ESM.docx]

**Additional file 4: Table S1** Primers used in this study.

| **Primer name** | **Sequence 5’—3’** | **Template** |
| --- | --- | --- |
| *ARR5-P1/P2* | CATGACCGGTGGAAACCAATAAAGCATATTTG | Col-4 genomic DNA |
|  | CATGCCATGGTGAGAGATGAGAGGAGAATAA |  |
| *AtABCG14-P1/P2* | ATGCCTCAGAACTGCATAGCAC | *rs1/rs2* genomic DNA |
|  | TTACCGCAACTTCACCCGATGC |  |
| 1-AC003979-2653 | CACTGCAACAAAGTGGAAAT | The genomic DNA of mutation plants isolated from F2 population |
|  | ATCCGTTTCAATATCCACAA |  |
| 1-AC006423-5434 | TGCGGGAGTGTGATAGAATA |  |
|  | TCCTCGAAAGATTCATTGAT |  |
| 1-AC000375-7539 | GAATTCTGTAACATCCCATTTCC |  |
|  | GGTCTAATTGCCGTTGTTGC |  |
| 1-AC010675-8645 | GGACCGACGGTTACGAGAGT |  |
|  | TAACGGGCCGTTGCAAGA |  |
| 2-AC007069-0197 | CGTGTTTACCGGGTCGGA |  |
|  | AAAACCCTTGAAGAATACG |  |
| 2-AC006420-1312 | TAGTCTGAGCTTACCAATA |  |
|  | TTACCCTCGACTCGTAAC |  |
| 2-AC005917-4269 | ATGTATTTGTTGCAAAATAA |  |
|  | TGCACAGAAGAAAAAACTA |  |
| 2-AC005623-5887 | TCCGATTCGATTAAACTC |  |
|  | TTATTTCCTATTTCAAGACT |  |
| 2-AC004697-8295 | ATGAACGGAGTAGCTATC |  |
|  | CGCGTAGAACATAATCTGTA |  |
| 3-AC009540-0422 | CAATGGGAAGAAGGTGTGAG |  |
|  | CGCATTTCCATAAGTTTGTT |  |
| 3-AB022217-2402 | ACCTGTTCAGTCTATGTTAC |  |
|  | GGGAATTATTAACATTATCA |  |
| 3-AB024028-4332 | ATGAGCTTTAGGAGTGTGTA |  |
|  | AATTTTGTCCCAAAAGAATA |  |
| 3-AC002534-6967 | CAAAAGAAATGCAACGAGAC |  |
|  | TTTGATCATGAATGGTAGTG |  |
| 3-AL132954-8728 | GAGCAACATTAAGGATAGAA |  |
|  | ATCTCATACTCATAATATGTAG |  |
| 4-AF069442-0706 | TTATAGCAAACGTACAAGTC |  |
|  | CTGCATACACGTCGTCTC |  |
| 4-AL049482-3304 | CTGGACCCTAGTGGATGT |  |
|  | GACGGTTCTCCATTAATTAT |  |
| 4-Z97343-5268 | TTCGGAGAAAGAAACGACAT |  |
|  | ATGGAACTATTCAGGCATTA |  |
| 4-AL078465-7217 | GCAACCGCTGCTGCTTTA |  |
|  | AATATTTGGCTTTGCGTAGA |  |
| 4-AL023094-8912 | ACCCTAAAACAATGTCTCTT |  |
|  | TGCTAACATGGAAATTTGTC |  |
| 5-AB010070-0918 | CTCTGTTGGGGCAAAACC |  |
|  | GATGCTGGAGAGTAGCTTAG |  |
| 5-AB006708-2862 | TTCATGAGAGCGGCATTC |  |
|  | GCAAAATGTTTGGACAATTA |  |
| 5-AF058826-5037 | CACAGGCCATTGGATGTA |  |
|  | TGTTAGAACCCACCATTTG |  |
| 5-AB023033-7443 | CCTGTTCCAATGAATATG |  |
|  | TGTAGCTGCTGAGTTGTC |  |
| 5-AB009053-9339 | AAAAGGCGACTACTAGCA |  |
|  | GCCATTTATTTGGTCAAC |  |
